# Effects of movement initiation pattern on dynamic postural stability across two individualized drop heights during single-leg landing in young men

**DOI:** 10.3389/fphys.2026.1973245

**Published:** 2026-10-21

**Authors:** Ying Li, Ruiqi Cheng, Idsart Kingma, Chris Bishop, Weijie Fu, Fei Li, Ming Li, Christophe Hautier, Qingshan Zhang

**Affiliations:** 1 School of Athletic Performance, Shanghai University of Sport, Shanghai, China; 2 Department of Human Movement Sciences, Faculty of Behavioural and Movement Sciences, Vrije Universiteit Amsterdam, Amsterdam Movement Sciences, Amsterdam, Netherlands; 3 Faculty of Science and Technology, London Sports Institute, Middlesex University, London, United Kingdom; 4 School of Intelligent Sports Engineering, Shanghai University of Sport, Shanghai, China; 5 Kunwei Sport Technology, Xinmin Vanke Superchain, No.305 Qixin Road, Minhang District, Shanghai, China; 6 Université Lyon 1, Laboratoire Interuniversitaire de Biologie de la Motricité – UR 7424, UFRSTAPS, Villeurbanne, France

**Keywords:** dynamic postural stability, dynamic postural stability index, ground reaction force, single leg landing, time to stabilization

## Abstract

**Objectives:**

Single-leg landing protocols differ in task components. We estimated pattern differences in vertical time to stabilization (V-TTS) and the Dynamic Postural Stability Index (DPSI) at two individualized heights, with 95% confidence intervals, and tested whether they changed with height.

**Methods:**

In a within-participant 3 × 2 factorial experiment, twenty-three healthy young male strength and conditioning students (5.7 ± 3.2 years of systematic sports training) completed randomized step-off (SOL), single-leg jump (SLJL), and single-leg hop (SLHL) landings at two individualized drop heights derived from countermovement jumps. Linear mixed-effects models tested pattern, height, and interaction effects on TTS and DPSI; statistical parametric mapping compared take-off and landing force waveforms.

**Results:**

Pattern × Height interactions occurred for V-TTS (p = 0.004) and DPSI (p = 0.001). At Low, the largest contrasts were SLHL-SOL for V-TTS (0.15 s; 95% CI, 0.11 to 0.20) and DPSI (0.079; 95% CI, 0.066 to 0.092; adjusted p < 0.001 for both). At High, both active patterns differed from SOL (V-TTS, 0.06-0.07 s; DPSI, 0.041-0.054; adjusted p ≤ 0.014) with no clear difference between the active patterns after adjustment. The SLHL–SOL and SLHL–SLJL differences for V-TTS and the SLJL–SOL and SLHL–SOL differences for DPSI were larger at Low, whereas the V-TTS SLJL–SOL and DPSI SLHL–SLJL contrasts showed no clear change. Horizontal TTS showed no adjusted differences. Take-off and landing interactions involved all force components.

**Conclusions:**

In healthy young men, movement initiation pattern altered V-TTS and DPSI at both heights, and some differences varied with height. Cross-study comparisons should match initiation pattern and prescribed height.

## Introduction

Non-contact lower limb injuries often occur during landing tasks, when the lower limb must attenuate impact loading and regain postural stability after foot contact (Dufek and Bates, 1990; McNitt-Gray, 1993; Belcher et al., 2024). Force plate-based single-leg landing tests are therefore widely used to quantify dynamic postural stability (DPS) from ground reaction force (GRF) after touchdown. Time to stabilization (TTS) estimates the time required for a GRF component to return to a steady-state threshold, whereas the Dynamic Postural Stability Index (DPSI) summarizes multidirectional GRF deviations during the post-landing period. These outcomes are commonly used in research on landing performance (Colby et al., 1999; Wikstrom et al., 2005a; Huurnink et al., 2019).

DPS has been assessed using several single-leg landing protocols, including hop-to-stabilization tasks, vertical jump landings, and single-leg drop landings (Wikstrom et al., 2005b; Sell, 2012; Malmir et al., 2019; García-Massó et al., 2019; Schmitz et al., 2019). The interpretation of these tests depends on whether the protocol provides comparable mechanical conditions (e.g., landing velocity, peak GRF, and impulse) across trials, participants, and studies (Afifi and Hinrichs, 2012; Gribble et al., 2012; McMahon et al., 2021). In practice, nominal task descriptions may not fully capture the mechanical conditions actually realized at touchdown. These conditions can be partially characterized by impact-related GRF characteristics, such as the early post-contact peak in vertical GRF. Because TTS and DPSI are computed from the GRF signal, such protocol-dependent force characteristics are directly relevant to their interpretation.

Drop height can alter the mechanical conditions at ground contact, including early post-contact GRF peaks and lower-limb energy-dissipation requirements, and may therefore influence GRF-derived DPS outcomes (Peng et al., 2019; Yeow et al., 2009). Movement initiation pattern may instead alter the pre-contact mechanics of landing. Previous biomechanical comparisons of step-off and jump-initiated landings have reported differences in pre-contact kinematics, vertical impulse, impact characteristics, and early GRF or muscle responses; however, these studies did not quantify initiation-related differences in vertical TTS (V-TTS) or DPSI (Afifi and Hinrichs, 2012; Harry et al., 2017; Pilanthananond et al., 2023). Prior DPS comparisons have generally contrasted complete landing tasks that differed in more than one respect, including height or travel distance, arm motion, or attentional demands, so the contribution of movement initiation could not be isolated (Wikstrom et al., 2005b; Schmitz et al., 2019). Consequently, the magnitude and uncertainty of initiation-related differences in V-TTS and DPSI remain unclear, as does whether those differences change between height conditions.

To address these questions, this study used a within-participant 3 × 2 factorial design to compare three movement initiation patterns—step-off landing (SOL), single-leg jump landing (SLJL), and single-leg hop landing (SLHL)—at two individualized drop heights (Low and High). The two actively propelled conditions also differed in propulsion direction and in the limb generating propulsion. The design did not compare the individualized heights with fixed absolute heights. The primary aims were to estimate the magnitude and 95% confidence intervals of pattern differences in V-TTS and DPSI within each height and to test whether those differences changed between heights. The overall height contrast was retained as a secondary estimate of the difference between the Low and High height conditions. Anteroposterior TTS (AP-TTS) and mediolateral TTS (ML-TTS) were analyzed as exploratory outcomes. Take-off and landing GRF waveforms were examined using statistical parametric mapping to locate when and in which force components task-related differences occurred. We hypothesized that (i) DPSI and V-TTS would be greater at High than at Low height; (ii) the active initiation patterns would produce greater DPSI and longer V-TTS than SOL; and (iii) Pattern × Height interactions would be present, such that relative pattern differences would not be consistent across the two height conditions.

## Materials and methods

### Participants

Twenty-three healthy young male strength and conditioning students (age = 20.8 ± 1.6 yrs, body mass = 74.41 ± 9.79 kg, height = 178.46 ± 5.48 cm, systematic sports training history = 5.67 ± 3.23 yrs) volunteered to participate. Participants were recruited from an available cohort of male strength and conditioning students, yielding a single-sex convenience sample. Given reported sex-related differences in dynamic postural stability and landing kinetics during single-leg landing (Wikstrom et al., 2006; Brown et al., 2009), protocol-related contrasts were evaluated within this common sex and training background. Eligibility required no lower-extremity musculoskeletal injury, surgery, or neurological disorder during the preceding 6 months.

An *a priori* G*Power analysis (ANOVA: repeated measures, within factors; Cohen’s f = 0.25 (Cohen, 1988), α = 0.05, power = 0.80, repeated-measures correlation = 0.50, nonsphericity correction ϵ = 1) indicated that 19 participants were required to detect a moderate within-subject effect across six repeated conditions (Verma and Verma, 2020). The final sample of 23 exceeded this planning-stage estimate. The institutional ethics committee approved this study on October 15, 2025 (approval no. 102772025RT187). All procedures complied with relevant laws and institutional guidelines and were conducted in accordance with the Declaration of Helsinki. All participants provided written informed consent before participation. Data were collected in a university biomechanics laboratory in December 2025.

### Experimental design and procedures

Participants performed single-leg landing tests on their dominant leg under a within-subject design comprising three movement initiation patterns and two individualized drop-height conditions, yielding six pattern × height conditions. The dominant leg was defined as the limb preferred for kicking a soccer ball (van Melick et al., 2017). The order of the six conditions was randomized separately for each participant before testing using a digital drawing-of-lots application.

The two individualized drop heights were defined from countermovement jump (CMJ) performance: the Low height was the mean of the left- and right-leg unilateral CMJ heights, and the High height was the bilateral CMJ height. The mean of both unilateral CMJ heights served as a participant-level reference for the Low height, while the dominant leg was used to standardize the landing side. After a standardized warm-up that included mobility, lower-body strength, and plyometric exercises, participants performed three maximal bilateral CMJs and three maximal unilateral CMJs per leg with the hands on the iliac crests. Jump height was calculated from flight time, and the three attempts were averaged for each CMJ task. Bilateral CMJ height was 47.3 ± 7.8 cm and unilateral CMJ height, averaged across limbs, was 22.1 ± 4.5 cm. Each participant’s CMJ-derived target height was matched to the nearest available box setting because box height could be adjusted only in discrete increments, giving box heights of 46.3 ± 7.4 cm in the High condition and 21.5 ± 3.7 cm in the Low condition. Mean absolute deviations from the individual target heights were 1.4 ± 1.1 cm for High and 1.3 ± 0.9 cm for Low. The realized ranges did not overlap, and the within-participant separation was 24.8 ± 4.9 cm. Three-dimensional ground reaction forces were sampled at 1000 Hz using two synchronized force platforms (KWYP FP6050; Kunwei, Shanghai, China); force platform 1 (FP1) was mounted on an adjustable jump box and force platform 2 (FP2) on the floor adjacent to FP1. The horizontal gap between FP1 and FP2 was adjusted once for each participant according to their preferred distance and then kept constant across all six conditions. Participants were instructed before testing to wear non-height-enhancing athletic shoes; shoe brand and model were not standardized.

From a quiet bipedal stance on FP1 with the hands on the iliac crests, participants descended onto FP2 using the assigned initiation pattern, landed on the dominant leg, and stabilized as quickly as possible into a quiet single-leg stance held for 20 s (Fransz et al., 2015). In step-off landing (SOL), the participant stepped forward off FP1 with the dominant limb without active vertical propulsion; the standardized cue was to “step forward off the box and land on one leg.” In single-leg hop landing (SLHL), the participant raised the nondominant limb, performed a submaximal vertical hop off FP1 on the dominant limb, and landed on the same limb; the cue was to “lift the front leg, hop off the box with the back leg, and land on the same leg.” In single-leg jump landing (SLJL), the dominant limb stepped forward toward FP2 while the nondominant limb remained on FP1 and provided submaximal forward propulsion; the cue was to “step the front leg forward, push off with the back leg, and land on the front leg.” For SLHL and SLJL, submaximal effort was instructed verbally, without prospective quantitative matching of propulsive impulse, take-off velocity, or jump height. Participants completed at least two familiarization trials for each movement initiation pattern at each height before recorded trials. A successful trial required the hands to remain on the iliac crests, a single-leg landing with no contralateral foot contact, and a quiet single-leg stance maintained for 20 s. Compliance with these criteria was monitored in real time. Recorded attempts continued until two successful trials were obtained in each condition, with a maximum of 10 attempts per condition. The first two successful trials were retained for analysis. Rest intervals were 30 s between attempts and 1 min between conditions.

### Data processing

All data processing was performed in MATLAB R2024a (MathWorks, Natick, MA, USA) using custom scripts. Raw GRF signals from each trial were segmented into a 20-s sequence beginning at initial contact on FP2, defined as the first instant at which vertical GRF exceeded 5% body weight (BW). This instant was set as t = 0 for landing-based analysis. BW was estimated from the quiet bilateral stance phase recorded on FP1 immediately before each trial.

For DPS analysis (TTS and DPSI), GRF were filtered using a zero-phase fourth-order low-pass Butterworth filter with a 12 Hz cutoff, consistent with prior DPS assessment methodology (Colby et al., 1999; Fransz et al., 2015; Ross and Guskiewicz, 2004). For statistical parametric mapping (SPM) analysis, the same filter order was applied with a 50 Hz cutoff to retain more of the force-time waveform structure. DPSI and its directional components (VSI, APSI, and MLSI) were calculated over the first 3 s after initial contact, following Wikstrom et al. (2005a). Component indices summarized root-mean-square GRF deviations over the first 3 s after contact: mediolateral and anteroposterior components were calculated relative to zero horizontal force, and the vertical component was calculated relative to body weight. DPSI was then calculated as the composite of these three component deviations and treated as a dimensionless GRF-based stability index. DPSI and its directional components were divided by body weight (N) after calculation, yielding dimensionless indices. Six published or previously described TTS algorithms were evaluated because TTS values and repeatability depend on calculation method and threshold definition (Fransz et al., 2015; Byrne et al., 2021). Fifteen prespecified algorithm-direction combinations were screened: SA, TOP1, and WAV in the V, AP, and ML directions, and RMS, TOP2, and TOP3 in the AP and ML directions only. The retained V-TTS outcome used the sequential averaging (SA) method, which defined stabilization as the time after which the cumulative average of the filtered V-GRF remained within trial mean ± 0.25 SD for the remainder of the 5-s post-contact window. The mean and SD were calculated over this window; no fixed minimum duration within the band was required. The retained AP-TTS and ML-TTS outcomes used TOP2, which applied an unbounded third-order polynomial to the absolute, mean-centered filtered horizontal GRF signal and defined TTS as the first sampled time at which the fitted curve fell below a baseline-variation threshold (calculation details in **Supplementary Methods S1**). Algorithm selection preceded the Pattern × Height models and was based solely on trial-to-trial repeatability; no estimate from the Pattern × Height models entered the selection. Complete candidate-algorithm definitions and repeatability outputs are reported in **Supplementary Tables S7** and **S8**.

Peak vertical GRF was defined as the maximum vertical GRF (V-GRF) recorded on FP2 during the first 100 ms after initial contact, computed from the V-GRF signal low-pass filtered at 50 Hz and normalized to BW. It was retained as an impact-related GRF descriptor of the landing, not as a DPS outcome.

### Statistical analysis

Linear mixed-effects models were used to analyze TTS and DPSI outcomes at the individual trial level. Fixed effects were movement initiation pattern (SOL, SLJL, SLHL), drop height (Low, High), and their interaction; participant was included as a random effect to account for repeated observations. Participant-specific random slopes for movement initiation pattern were evaluated for each outcome and were retained when models converged without singular fits and improved fit relative to the corresponding random-intercept model. Participant-specific random slopes for height were not included in the original candidate set; height and the Pattern × Height interaction were retained as fixed effects to estimate average within-participant height contrasts and their dependence on initiation pattern. The primary random structures are reported in **Supplementary Table S1**. Fixed-effect tests used Satterthwaite denominator degrees of freedom, followed by estimated marginal means and model-based contrasts. Model assumptions were evaluated using residual versus fitted plots and normal Q-Q plots, with Shapiro-Wilk tests used as supplementary checks of residual normality. When a Pattern × Height interaction was significant, pattern contrasts were evaluated separately within each height, with Bonferroni adjustment of p values within each three-contrast family. For the primary outcomes, three difference-of-differences contrasts per outcome additionally quantified whether each pattern difference changed between Low and High heights; these contrasts were defined as [(Pattern A − Pattern B) at Low] − [(Pattern A − Pattern B) at High]. Their p values were Bonferroni-adjusted within outcome, while the reported 95% confidence intervals were unadjusted. A marginal High–Low contrast collapsed across pattern was additionally retained as a secondary estimate. AP-TTS and ML-TTS were analyzed as exploratory outcomes.

Within-session between-trial repeatability used the intraclass correlation coefficient (ICC[2,1]), its 95% confidence interval (CI), and the standard error of measurement (SEM) (Koo and Li, 2016). The ICC[2,1] model was chosen to reflect single-trial repeatability within a session rather than the reliability of an averaged score. Pooled ICC[2,1] values were estimated across all participant × pattern × height targets for each outcome. For the four retained outcomes, percentile 95% CIs were obtained from 5,000 participant-level bootstrap samples, retaining all six targets and both trials from each sampled participant. Condition-specific SEM was calculated as the condition-specific trial-level SD multiplied by √(1 − pooled ICC[2,1]). This calculation used a common reliability coefficient across conditions and therefore assumed that the pooled ICC was applicable to each condition; these SEM values should be interpreted as approximations. Between-condition differences may increase between-target variance and the pooled ICC, which does not establish reliability within each condition separately. Reliability descriptors followed the lower bound of each 95% CI: poor (< 0.50), moderate (0.50-0.75), good (0.75-0.90), and excellent (> 0.90). Descriptive statistics are reported as mean ± SD with 95% CIs.

For SPM, take-off GRF waveforms were represented by the trial-specific interval from movement onset to take-off, whereas landing GRF waveforms comprised the first 400 ms after initial contact. For SOL, the take-off interval represented unloading and step-off rather than active propulsion. At 1000 Hz, each inclusive landing segment contained 401 samples. After normalization to BW, take-off and landing phases were separately time-normalized to 101 points (0-100%) using shape-preserving piecewise cubic interpolation and analyzed using two-way repeated-measures SPM ANOVA (movement initiation pattern × drop height) for each GRF component (Pataky et al., 2015). Both successful trials per condition were retained as replicate observations under the corresponding participant identifier in the omnibus models, yielding 276 waveforms per phase. *Post-hoc* two-sided paired nonparametric SPM (SnPM) comparisons with 5,000 permutations were conducted on each participant’s two-trial condition mean (Npair = 23): six pattern-within-height comparisons and three height-within-pattern comparisons. Bonferroni-adjusted thresholds were α = 0.0083 and α = 0.0167, respectively. Analyses were performed in MATLAB R2024a with spm1d M.0.4.52. Associations between peak vertical GRF and the primary DPS outcomes (DPSI and V-TTS) were tested with separate linear mixed-effects models (peak vertical GRF fixed, participant random); sensitivity models added movement initiation pattern, drop height, and their interaction to test whether the associations changed after adjustment. Scalar analyses were conducted in R (v4.4.2; R Core Team, Vienna, Austria) with α = 0.05.

## Results

Descriptive statistics and within-session between-trial repeatability for the four retained scalar outcomes are reported in **Table 1**. For each Pattern × Height condition, the two successful trials were first averaged within participant, and the resulting participant-level condition means were summarized across participants. Each condition included 23 participant-level condition means and 46 trial values. Pooled repeatability was moderate for V-TTS (ICC[2,1] = 0.77; 95% CI, 0.64 to 0.85), AP-TTS (ICC[2,1] = 0.81; 95% CI, 0.71 to 0.88), and ML-TTS (ICC[2,1] = 0.83; 95% CI, 0.72 to 0.89), and good for DPSI (ICC[2,1] = 0.92; 95% CI, 0.87 to 0.95). Model-estimated marginal means and 95% confidence intervals are shown in **Figure 1**. Complete candidate-algorithm definitions, screening results, and repeatability estimates are reported in **Supplementary Tables S7** and **S8**. Because ICCs were pooled across targets, SEM values in **Table 1** should be read as approximate condition-specific measurement-error descriptors, not as separate condition-level reliability tests or ICC estimates.

**Table 1:** Descriptive statistics and within-session between-trial repeatability for retained scalar DPS outcomes.

Outcome	Height	SOL mean ± SD	SOL SEM	SLJL mean ± SD	SLJL SEM	SLHL mean ± SD	SLHL SEM	Pooled ICC[2,1] (95% CI)
V-TTS	Low	1.82 ± 0.19	0.10	1.90 ± 0.17	0.08	1.98 ± 0.17	0.09	0.77 (0.64, 0.85)
V-TTS	High	1.90 ± 0.18	0.10	1.97 ± 0.13	0.07	1.96 ± 0.13	0.07	0.77 (0.64, 0.85)
DPSI	Low	0.236 ± 0.039	0.011	0.295 ± 0.047	0.014	0.315 ± 0.045	0.013	0.92 (0.87, 0.95)
DPSI	High	0.337 ± 0.051	0.015	0.378 ± 0.059	0.017	0.391 ± 0.051	0.015	0.92 (0.87, 0.95)
AP-TTS	Low	1.07 ± 1.05	0.46	1.09 ± 1.03	0.45	0.72 ± 0.89	0.41	0.81 (0.71, 0.88)
AP-TTS	High	1.20 ± 1.04	0.46	1.13 ± 0.92	0.42	1.10 ± 0.94	0.44	0.81 (0.71, 0.88)
ML-TTS	Low	2.77 ± 0.60	0.25	2.51 ± 0.61	0.25	2.65 ± 0.69	0.30	0.83 (0.72, 0.89)
ML-TTS	High	2.66 ± 0.74	0.31	2.66 ± 0.73	0.32	2.87 ± 0.71	0.31	0.83 (0.72, 0.89)

Values are mean ± SD across participant-level condition means. SEM values are condition-specific; pooled ICC[2,1] estimates were calculated across all Pattern × Height targets for each outcome. ICC confidence intervals were obtained from 5,000 participant-level bootstrap samples, retaining all six targets and both trials from each sampled participant. Each condition included 23 participants and 46 trial values. V-TTS was calculated using sequential averaging; AP-TTS and ML-TTS used TOP2. TTS values are reported in seconds; DPSI is dimensionless.

**Figure 1:**
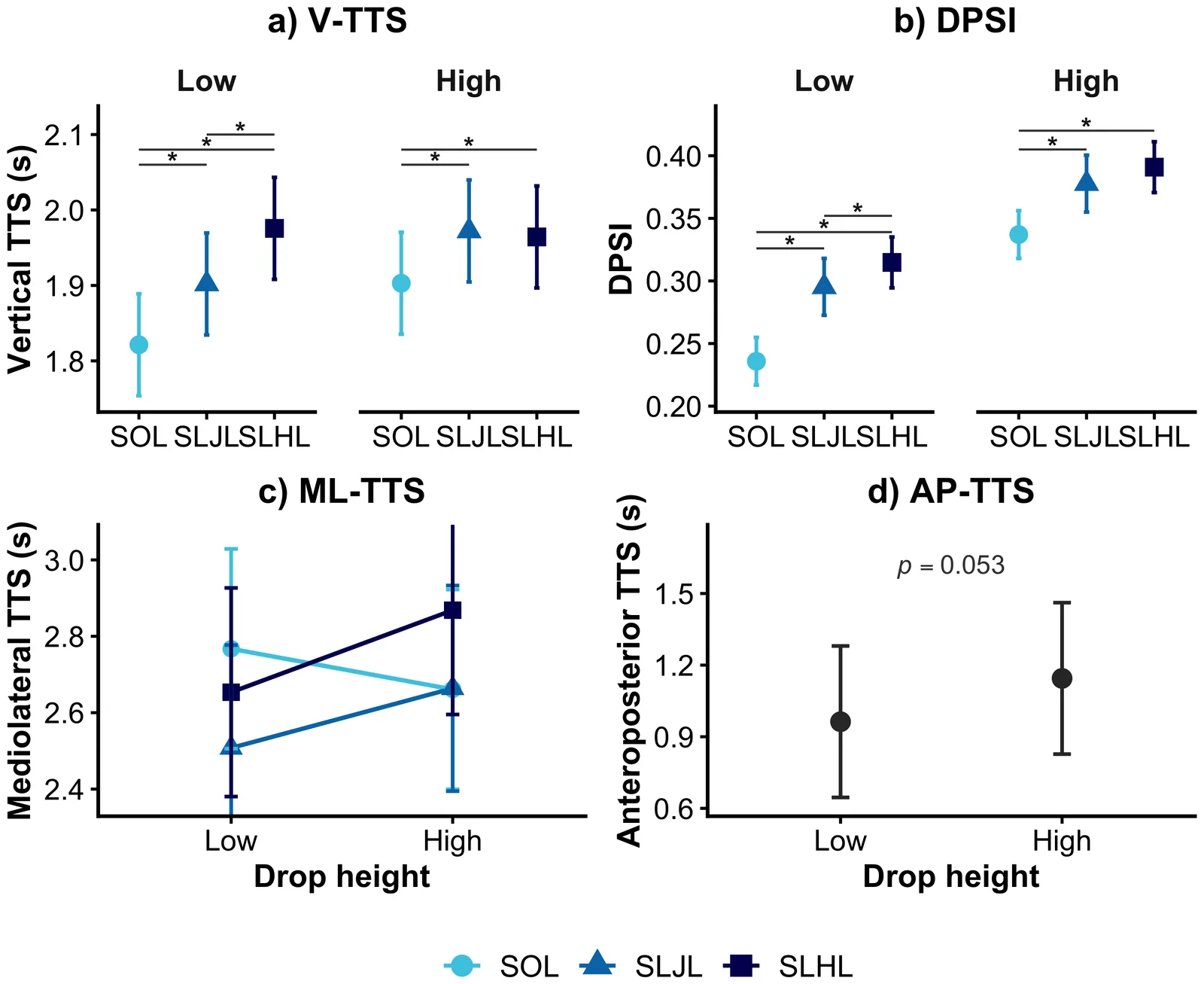
Estimated marginal means and 95% confidence intervals for TTS and DPSI outcomes across movement initiation patterns and height conditions. **(a, b)** show V-TTS and DPSI, respectively, with movement initiation patterns compared separately within each height condition. Horizontal lines with asterisks indicate Bonferroni-adjusted pairwise differences within the same height condition (p < 0.050). **(c)** shows ML-TTS without pairwise significance annotation because no Bonferroni-adjusted pairwise comparison was supported. **(d)** shows AP-TTS marginal estimated means collapsed across movement initiation patterns; the High-Low contrast was 0.18 s (95% CI, -0.003 to 0.366; p = 0.053). The pattern legend applies to **(a-c)**. SOL, step-off landing; SLJL, single-leg jump landing; SLHL, single-leg hop landing; TTS, time to stabilization; DPSI, Dynamic Postural Stability Index; V, vertical; ML, mediolateral; AP, anteroposterior.

### Vertical TTS and DPSI outcomes

Omnibus tests showed Pattern × Height interactions for V-TTS, F(2, 248) = 5.61, p = 0.004, and DPSI, F(2, 204) = 6.98, p = 0.001. Pattern contrasts were therefore examined within each height condition. At the Low height, all three pairwise pattern contrasts were significant for both outcomes. For V-TTS, SLJL was 0.081 s longer than SOL (95% CI, 0.038 to 0.123; adjusted p < 0.001), SLHL was 0.154 s longer than SOL (95% CI, 0.112 to 0.196; adjusted p < 0.001), and SLHL was 0.074 s longer than SLJL (95% CI, 0.032 to 0.116; adjusted p = 0.002). For DPSI, the corresponding Low-height differences were 0.059 (95% CI, 0.047 to 0.072; adjusted p < 0.001), 0.079 (95% CI, 0.066 to 0.092; adjusted p < 0.001), and 0.020 (95% CI, 0.007 to 0.032; adjusted p = 0.007). At the High height, SLJL and SLHL remained longer than SOL for V-TTS by 0.069 s (95% CI, 0.027 to 0.111; adjusted p = 0.004) and 0.061 s (95% CI, 0.019 to 0.104; adjusted p = 0.014), respectively, whereas SLHL and SLJL showed no adjusted pairwise difference (−0.008 s; 95% CI, −0.050 to 0.034; adjusted p = 1.000). Similarly, DPSI was greater for SLJL and SLHL than for SOL by 0.041 (95% CI, 0.028 to 0.054; adjusted p < 0.001) and 0.054 (95% CI, 0.041 to 0.067; adjusted p < 0.001), respectively, whereas the SLHL-SLJL contrast did not clearly differ after adjustment (0.013; 95% CI, 0.001 to 0.025; adjusted p = 0.103). Collapsed across movement initiation patterns, High height exceeded Low height by 0.047 s for V-TTS (95% CI, 0.022 to 0.071; p < 0.001) and by 0.087 for DPSI (95% CI, 0.081 to 0.092; p < 0.001). Complete condition-specific contrasts are reported in **Supplementary Table S3**.

For V-TTS, the SLHL–SOL difference was 0.093 s larger at Low height (95% CI, 0.033 to 0.153; Bonferroni-adjusted p = 0.007), and the SLHL–SLJL difference was 0.082 s larger at Low height (95% CI, 0.022 to 0.141; adjusted p = 0.023). There was no clear evidence that the SLJL–SOL difference changed between heights (0.011 s; 95% CI, −0.048 to 0.071; adjusted p = 1.000). For DPSI, the SLJL–SOL and SLHL–SOL differences were larger at Low height by 0.019 (95% CI, 0.005 to 0.033; adjusted p = 0.024) and 0.025 (95% CI, 0.011 to 0.039; adjusted p = 0.001), respectively. There was no clear evidence that the SLHL–SLJL difference changed between heights (0.006; 95% CI, −0.007 to 0.020; adjusted p = 1.000). Four of the six interaction contrasts showed clear evidence of larger pattern differences at Low; confidence intervals for the other two included zero, with all six contrasts reported in **Supplementary Table S3**.

### Exploratory horizontal TTS outcomes

AP-TTS and ML-TTS were interpreted as exploratory directional outcomes. AP-TTS showed no clear pattern, height, or interaction effect (F(2, 248) = 2.25, p = 0.108; F(1, 248) = 3.77, p = 0.053; F(2, 248) = 1.23, p = 0.294). No Bonferroni-adjusted marginal pattern contrast was significant: SLJL-SOL was −0.028 s (95% CI, −0.253 to 0.198; adjusted p = 1.000), SLHL-SOL was −0.223 s (95% CI, −0.448 to 0.003; adjusted p = 0.159), and SLHL-SLJL was −0.195 s (95% CI, −0.420 to 0.031; adjusted p = 0.270). The marginal High-Low estimate for AP-TTS was 0.181 s (95% CI, −0.003 to 0.366; p = 0.053). ML-TTS showed no clear pattern or height main effect (F(2, 22) = 1.55, p = 0.234; F(1, 204) = 2.43, p = 0.120). The Pattern × Height interaction was F(2, 204) = 3.05, p = 0.050, but none of the Bonferroni-adjusted within-height pattern contrasts was significant. At the Low height, the contrasts ranged from −0.260 to 0.146 s, with all confidence intervals including zero; at the High height, they ranged from 0.003 to 0.207 s, again with all adjusted contrasts showing no clear pairwise difference (**Supplementary Table S3**).

### SPM analysis

Take-off-phase SPM showed pattern main effects in all three GRF components and Pattern × Height interactions in V-GRF (60-89%), AP-GRF (0-2%, 3-6%, 7-15%, and 99-100%), and ML-GRF (27-32%). A height main effect was present for AP-GRF only. Pattern-within-height *post-hoc* comparisons localized the between-pattern differences (**Figure 2**). In V-GRF, SOL differed from both active patterns across broad portions of take-off at both heights, and SLJL differed from SLHL at the Low height from 78% to 92% of the phase. In AP-GRF, pattern contrasts were concentrated mainly during early-to-mid take-off and near the end of the normalized phase, whereas ML-GRF differences were smaller and more localized. Complete cluster-level locations, probabilities, extents, and comparison families are reported in **Supplementary Tables S4**-**S6**.

**Figure 2:**
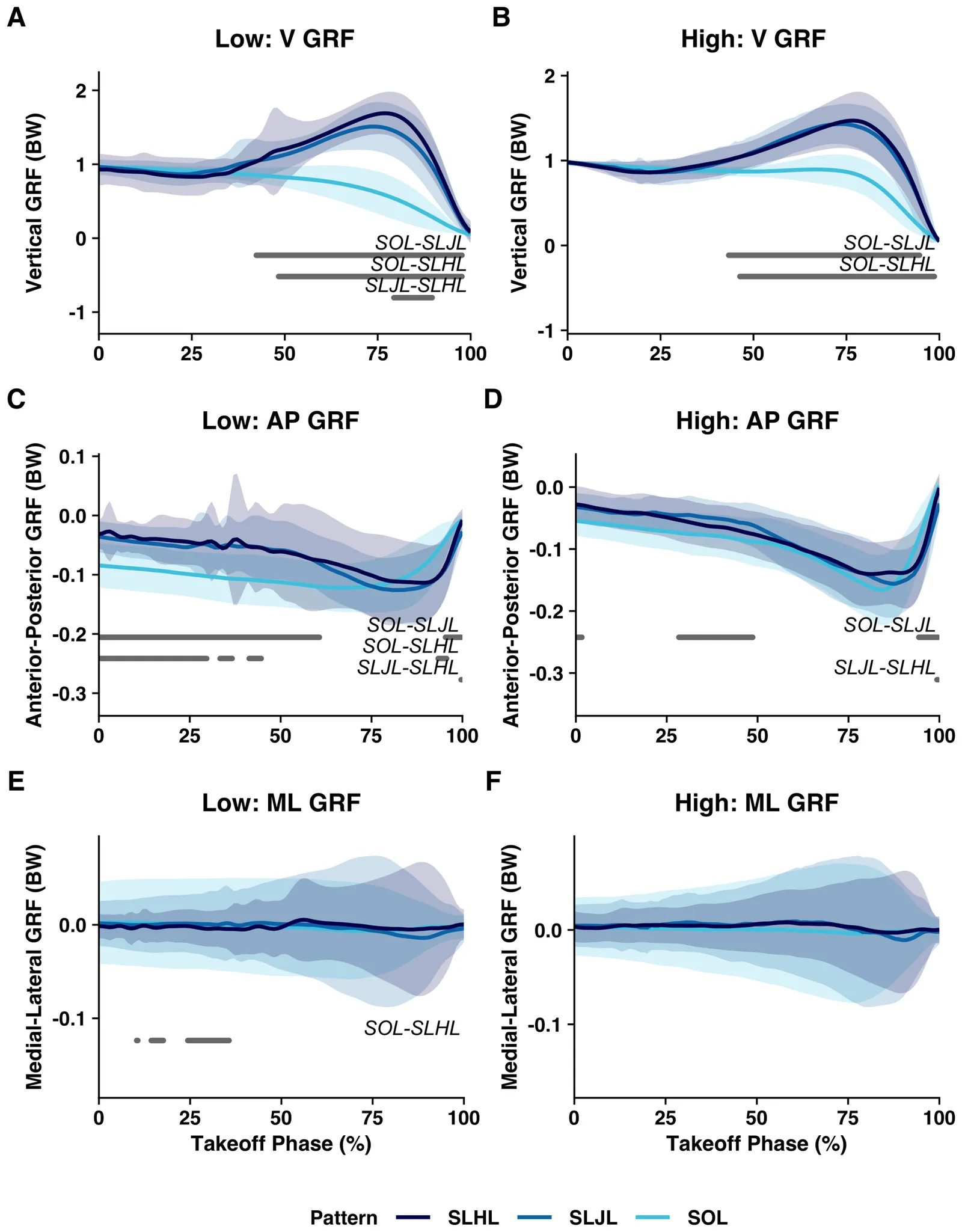
Take-off phase GRF waveforms (0-100%) for the three movement initiation patterns under the low and high height conditions. Take-off waveforms were time-normalized separately within each trial; because take-off duration varied between trials, equal percentages do not necessarily represent equal elapsed time. **(A, B)** show vertical (V), **(C, D)** anteroposterior (AP), and **(E, F)** mediolateral (ML) ground reaction force, with Low on the left and High on the right. All three components are displayed separately by height because each showed a significant Pattern × Height interaction. Solid curves are ensemble means and shaded bands are ±1 SD, computed by first averaging the two trials within each participant and condition and then averaging across the 23 participants. Horizontal bars indicate significant *post-hoc* SnPM clusters from pattern-within-height comparisons (Bonferroni-corrected α = 0.0083). Height-within-pattern comparisons are reported in the **Supplementary Material** and are not plotted. SOL, step-off landing; SLJL, single-leg jump landing; SLHL, single-leg hop landing; BW, body weight.

Landing-phase SPM showed pattern main effects and Pattern × Height interactions across all three GRF components. Interaction clusters occurred at 8-10%, 12-18%, and 36-41% for V-GRF; 1-5% and 13-16% for AP-GRF; and 14-16% for ML-GRF. Pattern-within-height waveform comparisons are shown in **Figure 3**. V-GRF pattern differences were present across all six pattern-within-height contrasts, indicating that the initiation patterns produced different vertical force-time profiles after contact at both prescribed heights. AP-GRF differences were also frequent but less uniformly distributed, whereas ML-GRF differences were brief and localized. These waveform findings were used to provide force-time context for the scalar DPS outcomes rather than to infer unmeasured joint kinematics or muscle activity. Complete cluster locations, probabilities, and pairwise results are reported in **Supplementary Tables S4**-**S6**. Associations between peak vertical GRF and the primary DPS outcomes are reported in **Supplementary Figure S1**.

**Figure 3:**
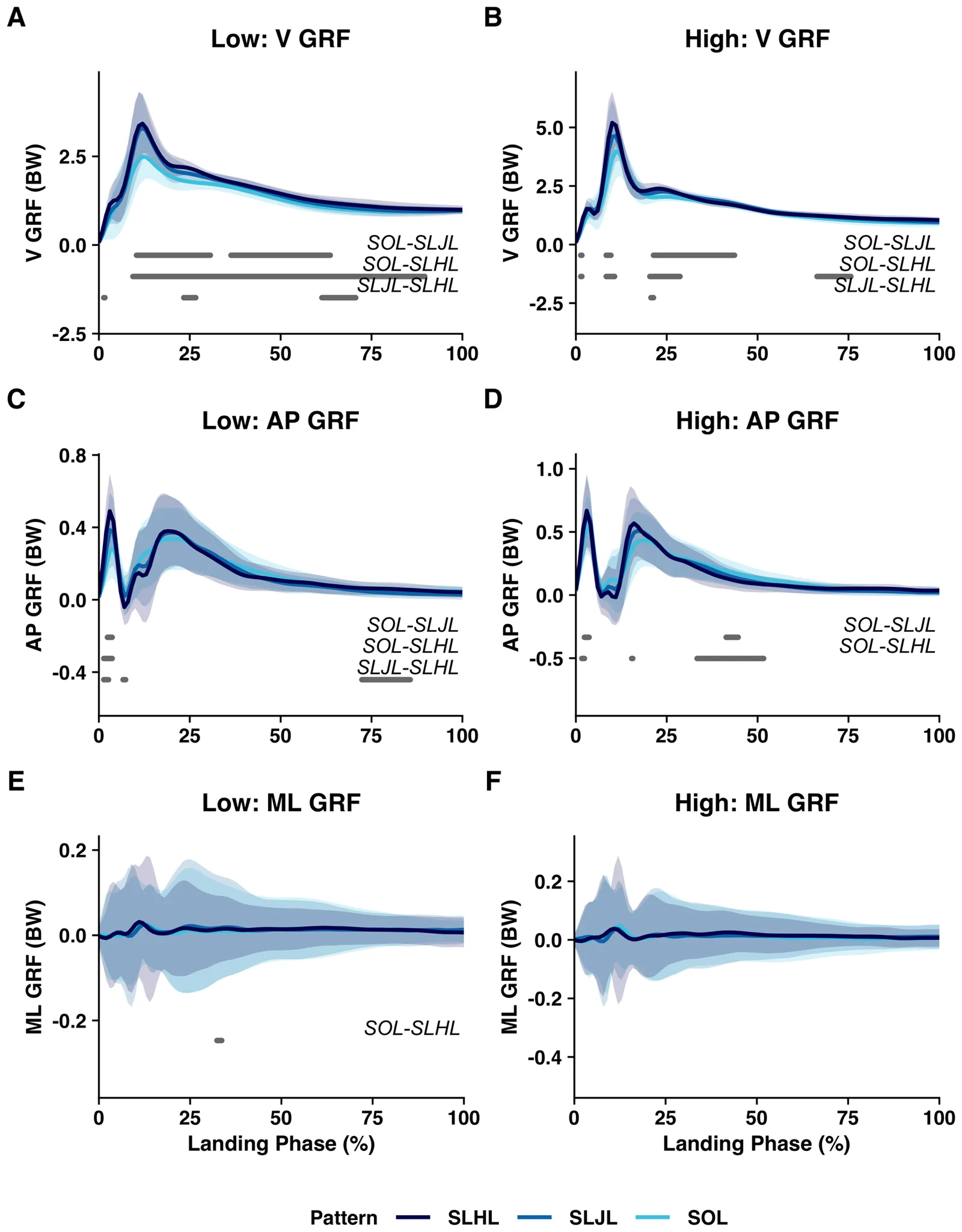
Landing phase GRF waveforms (0-100%) for the three movement initiation patterns under the low and high height conditions. For the landing phase, 0–100% represents the first 400 ms after initial ground contact. **(A, C, E)** show the Low condition and **(B, D, F)** show the High condition; vertical (V), anteroposterior (AP), and mediolateral (ML) components are arranged from top to bottom. Solid curves are ensemble means and shaded bands are ±1 SD, computed by first averaging the two trials within each participant and condition and then averaging across the 23 participants. Horizontal bars indicate significant *post-hoc* SnPM clusters from pattern-within-height comparisons (Bonferroni-corrected α = 0.0083). Height-within-pattern comparisons are reported in the **Supplementary Material** and are not plotted. SOL, step-off landing; SLJL, single-leg jump landing; SLHL, single-leg hop landing; BW, body weight.

In auxiliary peak vertical GRF models, peak V-GRF was associated with DPSI in the unadjusted participant-random model (β = 0.049 BW^-1^; 95% CI, 0.046 to 0.052; p < 0.001; marginal R² = 0.76, conditional R² = 0.86). Peak V-GRF was also associated with V-TTS in the corresponding unadjusted model (β = 0.019 s·BW^-1^; 95% CI, 0.006 to 0.032; p = 0.004; marginal R² = 0.02, conditional R² = 0.63). These R² values are model-specific summaries and were not statistically compared across outcomes. After adjustment for movement initiation pattern, height, and their interaction, the DPSI association remained positive (β = 0.030 BW^-1^; 95% CI, 0.025 to 0.034; p < 0.001), whereas the V-TTS association changed direction (β = −0.051 s·BW^-1^; 95% CI, −0.073 to −0.028; p < 0.001).

## Discussion

This study tested whether movement initiation pattern systematically affected V-TTS and DPSI at two individualized drop heights and whether these effects differed between the Low and High heights. Testing all six conditions within participants reduced confounding by stable between-participant differences and strengthened inference about the condition contrasts. Movement initiation pattern affected both V-TTS and DPSI within each height condition. However, the differences among patterns were not uniform across heights: some pairwise differences changed between the Low and High conditions, whereas others showed no clear evidence of change. Within the three initiation patterns and two height conditions examined, the primary evidence was provided by V-TTS and DPSI; the exploratory AP-TTS and ML-TTS analysis yielded no Bonferroni-adjusted pairwise differences among patterns.

At both prescribed heights, SOL differed from both active initiation patterns in V-TTS and DPSI. The tasks differed in propulsion direction and the limb generating propulsion; because propulsive intensity was instructed rather than quantitatively matched, the SLHL–SLJL contrast reflects the tasks as implemented rather than either feature alone. Previous step-off versus jump-initiated comparisons reported differences in pre-contact kinematics, vertical impulse, impact characteristics, and early GRF or muscle responses (Afifi and Hinrichs, 2012; Harry et al., 2017; Pilanthananond et al., 2023). These studies provide biomechanical plausibility for an initiation effect but do not directly address whether it is expressed in V-TTS or DPSI. Within the same two prescribed height conditions, landing limb, arm position, and stabilization instruction were held consistent across patterns. The present findings extend these earlier comparisons from pre-contact and early landing mechanics to V-TTS and DPSI. The observed differences may partly reflect initiation-specific mechanics rather than post-contact stabilization alone.

The Pattern × Height interactions showed that the SLHL–SOL and SLHL–SLJL differences in V-TTS and both SOL–active differences in DPSI were larger at Low than at High, whereas the remaining pairwise differences showed no clear evidence of change. The High-height V-TTS SLHL–SOL contrast was sensitive to the random-effects structure. Studies manipulating landing height have reported changes in landing kinetics, lower-limb muscle activation, and movement variability (Peng et al., 2019, 2011; Nordin and Dufek, 2017). Together, these studies show that height can alter landing mechanics and movement control, but they do not directly address whether differences among movement initiation patterns in V-TTS or DPSI also change with height. One possible interpretation is that the greater landing demand at the High height made some initiation-related differences less distinct in the resulting GRF-based outcomes, although this was not directly tested.

The SPM analysis complemented the scalar outcomes by locating task-related GRF differences across the take-off and landing phases and within individual force components. During take-off, Pattern × Height interactions were present in V-GRF, AP-GRF, and ML-GRF, showing that the force-time waveforms associated with the three initiation patterns differed before ground contact and that these differences varied across height conditions. For SOL, however, this interval represented unloading and stepping from the platform rather than active propulsion, so differences between SOL and the active patterns cannot be attributed to propulsion alone. Pattern-related waveform differences were also present during the landing phase and were distributed mainly across V-GRF and AP-GRF, with more limited differentiation in ML-GRF. SPM and the scalar DPS outcomes summarize different properties of the same GRF recordings: SPM locates waveform differences over normalized time, whereas V-TTS and DPSI reduce the post-contact signal to algorithm- and window-defined values. The waveforms therefore provide a force–time context for interpreting the scalar outcomes, but they do not reveal the joint movements or muscle activity underlying those differences.

The findings show that V-TTS and DPSI should be interpreted in relation to the movement initiation pattern and prescribed height condition. The descriptive condition means in **Supplementary Table S8** show that VSI was larger than APSI and MLSI and closely followed the pattern of DPSI across conditions. This pattern is consistent with a substantial contribution of vertical loading to the observed DPSI differences, but it does not establish a mechanism. Comparisons of complete protocols provide broader context by showing that TTS or DPSI can vary across protocols differing in several task features (Wikstrom et al., 2005b; Schmitz et al., 2019). The TTS results reported here are specific to the retained calculation definitions. Peak vertical GRF, reported in **Supplementary Figure S1**, was associated with DPSI. Because both were derived from the same GRF recording, this relationship should not be regarded as independent validation; Huurnink et al. (2019) reported a similar empirical relationship. The reversal of the peak V-GRF–V-TTS association after adjustment suggests that differences among pattern–height conditions contributed to the positive unadjusted association. The negative adjusted association may reflect a larger but more rapidly attenuated force peak, although force attenuation and neuromuscular mechanisms were not directly tested in this analysis. For repeated assessments and between-study comparisons, practitioners should standardize the movement initiation pattern, height-prescription method and actual box height, landing limb, footwear requirements, and propulsion, effort, and stabilization instructions. Signal processing and outcome calculations should also be consistent, including filtering, analysis windows, and the TTS algorithm, threshold, and stability criterion. Because the condition-specific SEM values were calculated using the pooled ICC and no minimal detectable change was established, small within-person changes should not be interpreted as definite improvement or deterioration.

Several limitations constrain interpretation. The small convenience sample limited estimate precision, and findings from healthy young men performing dominant-leg landings may not generalize to women, other age groups, injured populations, or nondominant-leg landings. Only three initiation patterns and two individualized drop heights were examined, and discrete box settings introduced small target deviations; the study therefore does not address other task or height constructions or compare individualized and fixed-height prescriptions. Propulsive intensity was instructed rather than impulse-matched, while SLHL and SLJL also differed in propulsion direction and the limb generating propulsion; the contrast therefore cannot be attributed to any single propulsive feature. GRF-only measurements could not identify kinematic or neuromuscular mechanisms, and two trials per condition limited individual-score precision and the stability of repeatability estimates. The normality and sphericity assumptions of the parametric SPM ANOVA were not formally assessed, so potential violations may affect the omnibus inference. Finally, selecting TTS definitions on the basis of repeatability in the same sample may have yielded optimistic repeatability estimates that require confirmation in independent data.

In healthy young men performing single-leg landings, changing how participants left the platform altered V-TTS and DPSI at both prescribed heights, and some pattern differences varied with height. Cross-study comparisons should therefore match movement initiation pattern and prescribed height.

## Data Availability

The raw data supporting the conclusions of this article will be made available by the authors, without undue reservation.
