# Supplementary material for: Effects of movement initiation pattern on dynamic postural stability across two individualized drop heights during single-leg landing in young men
Source: Front Physiol. 2026 Oct 21;17:1973245. doi: 10.3389/fphys.2026.1973245 (PMC13730876; doi:10.3389/fphys.2026.1973245)
Supplement: Supplementary file 1 [file SupplementaryFile1.docx]

Supplementary Material

This supplement provides calculation details and extended statistical outputs that are not repeated in the main manuscript. Updated estimated marginal means and model-based contrasts are reported in Supplementary Tables S2 and S3. Complete candidate-algorithm definitions and repeatability outputs are reported in Supplementary Tables S7 and S8.

# Supplementary Methods S1. Candidate TTS Algorithms and Screening

Six published or previously described time-to-stabilization (TTS) algorithms were screened before the final TTS variables were selected for analysis. The candidate set included sequential averaging (SA) [1], root mean square windowing (RMS) [2], three unbounded third-order polynomial variants (TOP1, TOP2, TOP3) [3,4], and a wavelet-based method (WAV) [5]. The broader rationale for screening multiple TTS methods was based on prior work showing that TTS values and reliability depend on calculation method, threshold definition, sampling rate, filtering, and trial length [6–8].

For TOP2, the standard reference windows were 10–15 s and 15–20 s after contact. Within each window, the window mean was subtracted from the filtered force, absolute values were taken, and the range was calculated. The smaller range was retained as the trial’s baseline variation. For each participant, task, initiation pattern, and height condition, the threshold was the mean plus 3 SD of these trial-level values, with a minimum of 1 N. For polynomial fitting, the force was expressed as its absolute deviation from the 10–20-s mean. Fitting extended from the largest deviation within the first 0.5 s after contact to 20 s after contact, or the recording end if earlier. TTS was the first sampled time at which the fitted curve fell below the threshold. As a numerical example, a 5-s SA record with a mean of 855.835 N and an SD of 202.879 N gave bounds of 805.115–906.555 N. The cumulative average was last outside the band at 1.864 s and remained within it thereafter, yielding a V-TTS of 1.864 s. In a separate TOP2 example, the fitted polynomial first fell below the 17.494-N threshold at 1.650 s, yielding an AP-TTS of 1.650 s.

Trial-to-trial repeatability for each algorithm-derived TTS variable was quantified using the intraclass correlation coefficient (ICC[2,1]), its 95% confidence interval (CI), and the standard error of measurement (SEM). Cases in which an algorithm failed to return a valid TTS were recorded. Reliability descriptors were assigned according to the lower bound of the 95% CI for each ICC estimate: poor (<0.50), moderate (0.50-0.75), good (0.75-0.90), and excellent (>0.90). For each parameter, the pooled ICC point estimate was calculated across complete participant × pattern × height target pairs. Its percentile 95% CI was obtained from 5,000 participant-level bootstrap samples, resampling participants with replacement and retaining all available complete targets and both trials from each sampled participant. Repeatability analyses and inference from scalar linear mixed-effects models used individual trials, whereas statistical parametric mapping (SPM) omnibus inference retained both trial waveforms under the corresponding participant identifier and post-hoc paired statistical nonparametric mapping (SnPM) comparisons used each participant's two-trial condition mean.

Fifteen algorithm–direction combinations were screened: SA, TOP1, and WAV in the V, AP, and ML directions, and RMS, TOP2, and TOP3 in the AP and ML directions only.

All 23 participants contributed data to every algorithm–direction combination. Fourteen of the 15 combinations yielded valid values for all 276 trials; the WAV AP calculation produced one capped 10.0-s return (0.4%), which was treated as invalid according to the defined stability criterion, leaving 275 valid trials and 137 complete trial pairs.

Algorithm screening retained SA for V-TTS (ICC = 0.77, 95% CI [0.64, 0.85]) and TOP2 for AP-TTS (ICC = 0.81, 95% CI [0.71, 0.88]) and ML-TTS (ICC = 0.83, 95% CI [0.72, 0.89]). DPSI showed a pooled ICC of 0.92 (95% CI [0.87, 0.95]). Descriptive statistics and condition-specific SEM values for the retained outcomes are reported in Table 1 of the main manuscript.

# Supplementary Methods S2. DPSI and Component Stability Indices

Dynamic postural stability was quantified from the ground reaction force (GRF) signal using the dynamic postural stability index (DPSI) and its directional component indices [9]. The component indices were calculated over the early landing analysis window as:

$$MLSI=\frac{1}{BW}\sqrt{\left[ \sum\frac{\left( 0-x \right)^{2}}{number of points} \right]}$$

$$APSI=\frac{1}{BW}\sqrt{\left[ \sum\frac{\left( 0-y \right)^{2}}{number of points} \right]}$$

$$VSI=\frac{1}{BW}\sqrt{\left[ \sum\frac{\left( BW-z \right)^{2}}{number of points} \right]}$$

where x, y, and z are the instantaneous mediolateral, anteroposterior, and vertical GRF samples, respectively, BW is body weight (N), and n is the number of samples in the first 3 s after initial contact. The composite DPSI was calculated as:

$$DPSI=\frac{1}{BW}\sqrt{\left[ \frac{\sum\left( 0-x \right)^{2}+\sum\left( 0-y \right)^{2}+\sum\left( BW-z \right)^{2}}{number of points} \right]}$$

APSI, MLSI, and VSI were retained as directional component indices for secondary analyses. DPSI was treated as the composite GRF-based stability index, whereas TTS variables represented the time required for each GRF component to return to a steady state threshold. All indices were divided by BW after calculation and are therefore dimensionless.

**Supplementary Methods S3. Statistical Details**

Scalar DPS outcomes, including TTS variables and DPSI, were analyzed at the individual trial level using linear mixed-effects model procedures in R. Linear mixed-effects models were fitted using restricted maximum likelihood with the lmerTest package. For each scalar outcome, the fixed-effect structure included movement initiation pattern, individualized drop height condition, and the Pattern × Height interaction. Participant was included as a random effect to account for repeated observations within participants.

A participant-specific random intercept was included in all models. A participant-specific random slope for movement initiation pattern was evaluated for each outcome and was retained only when the model converged without singular fits and improved model fit relative to the corresponding random intercept model. Participant-specific random slopes for height were not included in the original candidate set. Height and the Pattern × Height interaction were retained as fixed effects, so the models estimated average within-participant height contrasts but not participant-specific deviations in the height effect. As a sensitivity analysis, expanded models including random slopes for both Pattern and Height were fitted using the same data and fixed-effects structure. The expanded models converged without singular fits and had lower AIC values than the original models for all four outcomes. The primary Pattern × Height interaction directions were unchanged, although the precision of the High-height V-TTS SLHL–SOL contrast was reduced (Bonferroni-adjusted p = 0.057 in the expanded model versus p = 0.014 in the primary model). The primary random-effects structure for each outcome is summarized in Supplementary Table S1.

Fixed-effect tests used Satterthwaite denominator degrees of freedom. Estimated marginal means were obtained using the emmeans package. When a Pattern × Height interaction was significant, pairwise comparisons among movement initiation patterns were performed separately within each height condition. Each set of three pattern contrasts formed a family, and only p values were Bonferroni-adjusted; the reported two-sided 95% confidence intervals were unadjusted. For V-TTS and DPSI, three planned interaction contrasts per outcome additionally quantified whether each pattern difference changed between heights. These contrasts were defined as [(Pattern A − Pattern B) at Low] − [(Pattern A − Pattern B) at High], with Bonferroni-adjusted p values within outcome. A marginal High–Low contrast collapsed across pattern was additionally retained as a secondary estimate.

Model assumptions were evaluated using residual versus fitted plots and normal Q-Q plots, with Shapiro-Wilk tests used as a supplementary check of residual normality. Omnibus fixed-effect tests are reported with test statistics and p values. Estimated marginal means, standard errors, and 95% confidence intervals are provided in Supplementary Table S2. Supplementary Table S3 reports simple and marginal contrasts in Panel A and the six primary interaction contrasts in Panel B. Estimates are reported in original units with 95% confidence intervals and p values; interaction Hedges' g values were not calculated.

## Supplementary Table S1. Primary linear mixed model structure for scalar DPS outcomes.

| Outcome | Fixed effects | Primary random structure | Est. | df method | Post-hoc family |
| --- | --- | --- | --- | --- | --- |
| V-TTS | Pattern × Height | (1 \| Participant) | REML | Satterthwaite | Pattern within Height |
| DPSI | Pattern × Height | (1 + Pattern \| Participant) | REML | Satterthwaite | Pattern within Height |
| ML-TTS | Pattern × Height | (1 + Pattern \| Participant) | REML | Satterthwaite | Pattern within Height when interaction was significant |
| AP-TTS | Pattern × Height | (1 \| Participant) | REML | Satterthwaite | Marginal main-effect contrasts |

Note: Pattern refers to movement initiation pattern. REML = restricted maximum likelihood. The primary random structures were selected from the original candidate set of random-intercept and Pattern-slope models. Models additionally including Height random slopes were examined in the sensitivity analysis described in Supplementary Methods S3.

## Supplementary Table S2. Estimated marginal means and 95% confidence intervals for scalar DPS outcomes.

| **Outcome** | **Height** | **Pattern** | **EMM** | **SE** | **df** | **95% CI** |
| --- | --- | --- | --- | --- | --- | --- |
| V-TTS | Low | SOL | 1.821 | 0.033 | 32.1 | [1.754, 1.889] |
| V-TTS | Low | SLJL | 1.902 | 0.033 | 32.1 | [1.834, 1.970] |
| V-TTS | Low | SLHL | 1.976 | 0.033 | 32.1 | [1.908, 2.043] |
| V-TTS | High | SOL | 1.903 | 0.033 | 32.1 | [1.835, 1.971] |
| V-TTS | High | SLJL | 1.972 | 0.033 | 32.1 | [1.905, 2.040] |
| V-TTS | High | SLHL | 1.964 | 0.033 | 32.1 | [1.897, 2.032] |
| DPSI | Low | SOL | 0.236 | 0.009 | 25.5 | [0.217, 0.255] |
| DPSI | Low | SLJL | 0.295 | 0.011 | 24.4 | [0.273, 0.318] |
| DPSI | Low | SLHL | 0.315 | 0.010 | 25.0 | [0.295, 0.335] |
| DPSI | High | SOL | 0.337 | 0.009 | 25.5 | [0.318, 0.356] |
| DPSI | High | SLJL | 0.378 | 0.011 | 24.4 | [0.355, 0.400] |
| DPSI | High | SLHL | 0.391 | 0.010 | 25.0 | [0.371, 0.411] |
| ML-TTS | Low | SOL | 2.767 | 0.128 | 30.1 | [2.506, 3.029] |
| ML-TTS | Low | SLJL | 2.508 | 0.132 | 29.5 | [2.239, 2.777] |
| ML-TTS | Low | SLHL | 2.654 | 0.134 | 29.3 | [2.380, 2.927] |
| ML-TTS | High | SOL | 2.661 | 0.128 | 30.1 | [2.399, 2.922] |
| ML-TTS | High | SLJL | 2.664 | 0.132 | 29.5 | [2.395, 2.933] |
| ML-TTS | High | SLHL | 2.868 | 0.134 | 29.3 | [2.595, 3.141] |
| AP-TTS | Low | Collapsed across Pattern | 0.963 | 0.155 | 26.6 | [0.646, 1.280] |
| AP-TTS | High | Collapsed across Pattern | 1.144 | 0.155 | 26.6 | [0.827, 1.462] |

Note: EMM = estimated marginal mean; SE = standard error; CI = confidence interval. V-TTS, AP-TTS, and ML-TTS are reported in seconds; DPSI is dimensionless.

## Supplementary Table S3. Model-based contrasts for scalar DPS outcomes.

**Panel A. Simple and marginal contrasts.**

| **Outcome** | **Contrast family / context** | **Contrast** | **Estimate** | **SE** | **df** | **95% CI** | **p** |
| --- | --- | --- | --- | --- | --- | --- | --- |
| V-TTS | Pattern within Height: Low | SLJL - SOL | 0.081 | 0.021 | 248.00 | [0.038, 0.123] | <0.001 |
| V-TTS | Pattern within Height: Low | SLHL - SOL | 0.154 | 0.021 | 248.00 | [0.112, 0.196] | <0.001 |
| V-TTS | Pattern within Height: Low | SLHL - SLJL | 0.074 | 0.021 | 248.00 | [0.032, 0.116] | 0.002 |
| V-TTS | Pattern within Height: High | SLJL - SOL | 0.069 | 0.021 | 248.00 | [0.027, 0.111] | 0.004 |
| V-TTS | Pattern within Height: High | SLHL - SOL | 0.061 | 0.021 | 248.00 | [0.019, 0.104] | 0.014 |
| V-TTS | Pattern within Height: High | SLHL - SLJL | -0.008 | 0.021 | 248.00 | [-0.050, 0.034] | 1.000 |
| V-TTS | Marginal Height: Collapsed across Pattern | High - Low | 0.047 | 0.012 | 248.00 | [0.022, 0.071] | <0.001 |
| DPSI | Pattern within Height: Low | SLJL - SOL | 0.059 | 0.006 | 43.83 | [0.047, 0.072] | <0.001 |
| DPSI | Pattern within Height: Low | SLHL - SOL | 0.079 | 0.007 | 42.66 | [0.066, 0.092] | <0.001 |
| DPSI | Pattern within Height: Low | SLHL - SLJL | 0.020 | 0.006 | 48.34 | [0.007, 0.032] | 0.007 |
| DPSI | Pattern within Height: High | SLJL - SOL | 0.041 | 0.006 | 43.83 | [0.028, 0.054] | <0.001 |
| DPSI | Pattern within Height: High | SLHL - SOL | 0.054 | 0.007 | 42.66 | [0.041, 0.067] | <0.001 |
| DPSI | Pattern within Height: High | SLHL - SLJL | 0.013 | 0.006 | 48.34 | [0.001, 0.025] | 0.103 |
| DPSI | Marginal Height: Collapsed across Pattern | High - Low | 0.087 | 0.003 | 204.00 | [0.081, 0.092] | <0.001 |
| ML-TTS | Pattern within Height: Low | SLJL - SOL | -0.260 | 0.144 | 36.80 | [-0.552, 0.033] | 0.240 |
| ML-TTS | Pattern within Height: Low | SLHL - SOL | -0.114 | 0.137 | 39.24 | [-0.391, 0.163] | 1.000 |
| ML-TTS | Pattern within Height: Low | SLHL - SLJL | 0.146 | 0.122 | 46.99 | [-0.099, 0.390] | 0.710 |
| ML-TTS | Pattern within Height: High | SLJL - SOL | 0.003 | 0.144 | 36.80 | [-0.289, 0.295] | 1.000 |
| ML-TTS | Pattern within Height: High | SLHL - SOL | 0.207 | 0.137 | 39.24 | [-0.069, 0.484] | 0.413 |
| ML-TTS | Pattern within Height: High | SLHL - SLJL | 0.205 | 0.122 | 46.99 | [-0.040, 0.449] | 0.298 |
| AP-TTS | Marginal Pattern: Collapsed across Height | SLJL - SOL | -0.028 | 0.114 | 248.00 | [-0.253, 0.198] | 1.000 |
| AP-TTS | Marginal Pattern: Collapsed across Height | SLHL - SOL | -0.223 | 0.114 | 248.00 | [-0.448, 0.003] | 0.159 |
| AP-TTS | Marginal Pattern: Collapsed across Height | SLHL - SLJL | -0.195 | 0.114 | 248.00 | [-0.420, 0.031] | 0.270 |
| AP-TTS | Marginal Height: Collapsed across Pattern | High - Low | 0.181 | 0.093 | 248.00 | [-0.003, 0.366] | 0.053 |

**Panel B. Primary Pattern × Height interaction contrasts.**

| **Outcome** | **Pattern contrast** | **Estimate** | **SE** | **df** | **95% CI** | **Bonferroni-adjusted p** |
| --- | --- | --- | --- | --- | --- | --- |
| V-TTS | SLJL - SOL | 0.011 | 0.030 | 248 | [-0.048, 0.071] | 1.000 |
| V-TTS | SLHL - SOL | 0.093 | 0.030 | 248 | [0.033, 0.153] | 0.007 |
| V-TTS | SLHL - SLJL | 0.082 | 0.030 | 248 | [0.022, 0.141] | 0.023 |
| DPSI | SLJL - SOL | 0.019 | 0.007 | 204 | [0.005, 0.033] | 0.024 |
| DPSI | SLHL - SOL | 0.025 | 0.007 | 204 | [0.011, 0.039] | 0.001 |
| DPSI | SLHL - SLJL | 0.006 | 0.007 | 204 | [-0.007, 0.020] | 1.000 |

*Note: In Panel A, p values were Bonferroni-adjusted within each three-contrast family; single marginal-height contrasts were unadjusted. Panel B estimates equal [(Pattern A - Pattern B) at Low] - [(Pattern A - Pattern B) at High], with positive values indicating a larger Low-height difference; p values were Bonferroni-adjusted within outcome. All 95% confidence intervals are unadjusted. TTS estimates are in seconds; DPSI is dimensionless. Interaction Hedges' g was not calculated.*

# Supplementary Methods S4. SPM Analysis Procedure

The take-off phase was represented by the trial-specific interval from movement onset to take-off, whereas the landing phase comprised the first 400 ms after initial contact. At 1000 Hz, each inclusive landing segment contained 401 samples. After normalization to BW, both phases were resampled separately to 101 points (0–100%) using shape-preserving piecewise cubic interpolation. For each GRF component, a two-way repeated-measures SPM ANOVA tested movement initiation pattern, height, and their interaction. Both successful trials per condition were retained as replicate observations under the corresponding participant identifier in the omnibus models, yielding 276 waveforms per phase. No formal tests of residual normality or sphericity were performed for the SPM ANOVA, and no correction for nonsphericity was applied.

Post-hoc two-sided paired SnPM comparisons with 5,000 permutations were conducted on each participant's two-trial condition mean (Npair = 23) for every phase and component. All six phase-by-component omnibus analyses showed a Pattern × Height interaction; pattern-within-height comparisons therefore formed the primary post-hoc family for every component.

Pairwise post-hoc waveform comparisons were organized into two predefined families: six pattern-within-height comparisons and three height-within-pattern comparisons. Bonferroni adjustment yielded α = 0.0083 and α = 0.0167, respectively. Pattern-within-height comparisons formed the primary family displayed in Figures 2 and 3; height-within-pattern comparisons were retained as transparency checks and were not plotted. Analyses were performed in MATLAB R2024a with spm1d M.0.4.52.

**Supplementary Table S4. SPM ANOVA cluster summary for take-off and landing phases.**

| **Phase** | **GRF component** | **Effect** | **Cluster location** | **Cluster p** | **Extent** |
| --- | --- | --- | --- | --- | --- |
| Take-off | V-GRF | Pattern | 39–99% | <0.001 | 59.500 |
| Take-off | V-GRF | Pattern × Height | 60–89% | <0.001 | 28.500 |
| Take-off | AP-GRF | Pattern | 0–57% | <0.001 | 56.300 |
| Take-off | AP-GRF | Pattern | 92–100% | <0.001 | 7.700 |
| Take-off | AP-GRF | Height | 0–2% | 0.04500 | 1.200 |
| Take-off | AP-GRF | Height | 4–9% | 0.02000 | 3.500 |
| Take-off | AP-GRF | Height | 10–14% | 0.02900 | 2.700 |
| Take-off | AP-GRF | Height | 79–98% | <0.001 | 18.300 |
| Take-off | AP-GRF | Pattern × Height | 0–2% | 0.03800 | 1.900 |
| Take-off | AP-GRF | Pattern × Height | 3–6% | 0.04100 | 1.600 |
| Take-off | AP-GRF | Pattern × Height | 7–15% | 0.00100 | 7.100 |
| Take-off | AP-GRF | Pattern × Height | 99–100% | 0.04700 | 0.900 |
| Take-off | ML-GRF | Pattern | 0–42% | <0.001 | 41.400 |
| Take-off | ML-GRF | Pattern × Height | 27–32% | 0.02900 | 3.500 |
| Landing | V-GRF | Pattern | 0–3% | 0.02100 | 2.700 |
| Landing | V-GRF | Pattern | 6–15% | <0.001 | 7.800 |
| Landing | V-GRF | Pattern | 17–90% | <0.001 | 72.300 |
| Landing | V-GRF | Height | 0–14% | <0.001 | 13.600 |
| Landing | V-GRF | Height | 23–84% | <0.001 | 59.700 |
| Landing | V-GRF | Pattern × Height | 8–10% | 0.04996 | 0.100 |
| Landing | V-GRF | Pattern × Height | 12–18% | 0.00300 | 5.000 |
| Landing | V-GRF | Pattern × Height | 36–41% | 0.00800 | 4.000 |
| Landing | AP-GRF | Pattern | 0–5% | 0.00800 | 4.600 |
| Landing | AP-GRF | Pattern | 5–8% | 0.03400 | 2.000 |
| Landing | AP-GRF | Pattern | 15–19% | 0.02300 | 3.000 |
| Landing | AP-GRF | Pattern | 31–51% | <0.001 | 19.400 |
| Landing | AP-GRF | Pattern | 82–90% | <0.001 | 7.400 |
| Landing | AP-GRF | Height | 0–6% | 0.00300 | 5.500 |
| Landing | AP-GRF | Height | 9–12% | 0.03900 | 1.600 |
| Landing | AP-GRF | Height | 14–24% | <0.001 | 9.300 |
| Landing | AP-GRF | Height | 99–100% | 0.04996 | 0.100 |
| Landing | AP-GRF | Pattern × Height | 1–5% | 0.01800 | 3.400 |
| Landing | AP-GRF | Pattern × Height | 13–16% | 0.03100 | 2.300 |
| Landing | ML-GRF | Pattern | 9–12% | 0.04300 | 1.400 |
| Landing | ML-GRF | Pattern | 25–34% | <0.001 | 7.400 |
| Landing | ML-GRF | Pattern | 38–46% | 0.00100 | 6.900 |
| Landing | ML-GRF | Height | 10–12% | 0.04987 | 0.200 |
| Landing | ML-GRF | Pattern × Height | 14–16% | 0.04800 | 0.700 |

Note: Cluster-level p values are supra-threshold cluster probabilities from the SPM ANOVA. Post-hoc Bonferroni thresholds do not apply to these omnibus clusters. Extent is reported in normalized phase units.

Supplementary Table S5. SPM post-hoc cluster summary for take-off and landing phases.

The post-hoc analysis yielded 70 supra-threshold clusters across the take-off and landing phases. A count summary by phase, GRF component, and comparison family is provided below; complete cluster locations, supra-threshold p values, extents, and pair counts are reported in Supplementary Table S6.

| **Phase** | **GRF component** | **Post-hoc family** | **Number of clusters** |
| --- | --- | --- | --- |
| Take-off | V-GRF | Pattern within height | 5 |
| Take-off | V-GRF | Height within pattern | 2 |
| Take-off | AP-GRF | Pattern within height | 11 |
| Take-off | AP-GRF | Height within pattern | 4 |
| Take-off | ML-GRF | Pattern within height | 3 |
| Take-off | ML-GRF | Height within pattern | 1 |
| Landing | V-GRF | Pattern within height | 15 |
| Landing | V-GRF | Height within pattern | 8 |
| Landing | AP-GRF | Pattern within height | 11 |
| Landing | AP-GRF | Height within pattern | 9 |
| Landing | ML-GRF | Pattern within height | 1 |
| Landing | ML-GRF | Height within pattern | 0 |

Note: Cluster-level p values are supra-threshold cluster probabilities from paired SnPM comparisons. All comparisons used Npair = 23. Height codes are reported as High and Low, and movement initiation patterns are abbreviated as SOL (step-off landing), SLJL (single-leg jump landing), and SLHL (single-leg hop landing). Pattern-within-height comparisons form the primary post-hoc family and are displayed in Figures 2 and 3; height-within-pattern comparisons are reported here as a transparency check and are not plotted.

# Supplementary Methods S5. Task Instructions, Familiarization, and Trial Acceptance

Standardized verbal cues were used for each movement initiation pattern. SOL: "step forward off the box and land on one leg." SLHL: "lift the front leg, hop off the box with the back leg, and land on the same leg." SLJL: "step the front leg forward while the back leg pushes off, and land on the front leg." Submaximal effort in SLHL and SLJL was instructed verbally but was not quantitatively monitored or matched. Before recorded trials, participants performed at least two familiarization trials for each movement initiation pattern at each height condition to ensure that they understood the task requirements.

A successful trial required the hands to remain on the iliac crests, a single-leg landing with no contralateral foot contact, and a quiet single-leg stance maintained for 20 s. The investigator checked these criteria during testing. The first two successful trials per condition were retained, with a maximum of 10 attempts allowed per condition.

**Supplementary Methods S6. CMJ Testing and Height Prescription**

Individualized drop heights were used to scale the landing task to each participant's jump capacity and preserve ecological relevance of the task demands, rather than to test the effect of individualization itself. After a standardized warm-up including mobility, lower body strength, and plyometric exercises, participants performed three maximal bilateral CMJs and three maximal unilateral CMJs per leg, with the hands on the iliac crests. Jump height was calculated from flight time and averaged across the three trials for the bilateral CMJ and for each unilateral CMJ. Mean absolute deviations of the realized box heights from the individual targets were 1.4 ± 1.1 cm (High) and 1.3 ± 0.9 cm (Low).

## Supplementary Table S6. Complete SPM post-hoc cluster-level results for take-off and landing phases.

| **Phase** | **GRF component** | **Comparison** | **Height/context** | | | **Cluster location** | **Cluster p** | **Extent** | **Npair** | | **Post-hoc family** |
| --- | --- | --- | --- | --- | --- | --- | --- | --- | --- | --- | --- |
| Take-off | V-GRF | SLJL vs SLHL | | Low | 78–92% | | <0.001 | 13.000 | 23 | Pattern within height | |
| Take-off | V-GRF | SOL vs SLHL | | Low | 47–99% | | <0.001 | 50.600 | 23 | Pattern within height | |
| Take-off | V-GRF | SOL vs SLJL | | Low | 41–99% | | <0.001 | 57.100 | 23 | Pattern within height | |
| Take-off | V-GRF | SOL vs SLHL | | High | 45–100% | | <0.001 | 53.300 | 23 | Pattern within height | |
| Take-off | V-GRF | SOL vs SLJL | | High | 42–96% | | <0.001 | 53.200 | 23 | Pattern within height | |
| Take-off | V-GRF | High vs Low | | Within SOL | 57–92% | | <0.001 | 34.300 | 23 | Height within pattern | |
| Take-off | V-GRF | High vs Low | | Within SOL | 96–100% | | 0.00300 | 2.200 | 23 | Height within pattern | |
| Take-off | AP-GRF | SLJL vs SLHL | | Low | 99–100% | | 0.00300 | 0.400 | 23 | Pattern within height | |
| Take-off | AP-GRF | SOL vs SLHL | | Low | 0–31% | | <0.001 | 30.300 | 23 | Pattern within height | |
| Take-off | AP-GRF | SOL vs SLHL | | Low | 32–38% | | <0.001 | 5.300 | 23 | Pattern within height | |
| Take-off | AP-GRF | SOL vs SLHL | | Low | 40–46% | | <0.001 | 5.300 | 23 | Pattern within height | |
| Take-off | AP-GRF | SOL vs SLHL | | Low | 51–53% | | 0.00500 | 0.300 | 23 | Pattern within height | |
| Take-off | AP-GRF | SOL vs SLHL | | Low | 92–98% | | 0.00100 | 4.600 | 23 | Pattern within height | |
| Take-off | AP-GRF | SOL vs SLJL | | Low | 0–62% | | <0.001 | 61.800 | 23 | Pattern within height | |
| Take-off | AP-GRF | SOL vs SLJL | | Low | 94–100% | | <0.001 | 5.900 | 23 | Pattern within height | |
| Take-off | AP-GRF | SLJL vs SLHL | | High | 97–100% | | <0.001 | 2.100 | 23 | Pattern within height | |
| Take-off | AP-GRF | SOL vs SLJL | | High | 29–50% | | <0.001 | 19.600 | 23 | Pattern within height | |
| Take-off | AP-GRF | SOL vs SLJL | | High | 93–100% | | <0.001 | 6.100 | 23 | Pattern within height | |
| Take-off | AP-GRF | High vs Low | | Within SLJL | 91–98% | | <0.001 | 6.100 | 23 | Height within pattern | |
| Take-off | AP-GRF | High vs Low | | Within SOL | 0–51% | | <0.001 | 50.300 | 23 | Height within pattern | |
| Take-off | AP-GRF | High vs Low | | Within SOL | 76–95% | | <0.001 | 18.600 | 23 | Height within pattern | |
| Take-off | AP-GRF | High vs Low | | Within SOL | 99–100% | | 0.00820 | 0.009 | 23 | Height within pattern | |
| Take-off | ML-GRF | SOL vs SLHL | | Low | 9–11% | | 0.00520 | 0.009 | 23 | Pattern within height | |
| Take-off | ML-GRF | SOL vs SLHL | | Low | 14–18% | | 0.00200 | 3.600 | 23 | Pattern within height | |
| Take-off | ML-GRF | SOL vs SLHL | | Low | 24–37% | | <0.001 | 11.100 | 23 | Pattern within height | |
| Take-off | ML-GRF | High vs Low | | Within SLHL | 27–32% | | 0.00200 | 4.900 | 23 | Height within pattern | |
| Landing | V-GRF | SLJL vs SLHL | | Low | 0–3% | | 0.00100 | 1.400 | 23 | Pattern within height | |
| Landing | V-GRF | SLJL vs SLHL | | Low | 22–28% | | <0.001 | 4.800 | 23 | Pattern within height | |
| Landing | V-GRF | SLJL vs SLHL | | Low | 60–72% | | <0.001 | 11.000 | 23 | Pattern within height | |
| Landing | V-GRF | SOL vs SLHL | | Low | 8–91% | | <0.001 | 82.400 | 23 | Pattern within height | |
| Landing | V-GRF | SOL vs SLJL | | Low | 9–31% | | <0.001 | 21.300 | 23 | Pattern within height | |
| Landing | V-GRF | SOL vs SLJL | | Low | 36–65% | | <0.001 | 28.100 | 23 | Pattern within height | |
| Landing | V-GRF | SLJL vs SLHL | | High | 20–22% | | 0.00400 | 0.200 | 23 | Pattern within height | |
| Landing | V-GRF | SOL vs SLHL | | High | 0–3% | | <0.001 | 2.100 | 23 | Pattern within height | |
| Landing | V-GRF | SOL vs SLHL | | High | 7–12% | | <0.001 | 3.800 | 23 | Pattern within height | |
| Landing | V-GRF | SOL vs SLHL | | High | 19–29% | | <0.001 | 9.100 | 23 | Pattern within height | |
| Landing | V-GRF | SOL vs SLHL | | High | 66–75% | | 0.00200 | 7.800 | 23 | Pattern within height | |
| Landing | V-GRF | SOL vs SLJL | | High | 0–3% | | <0.001 | 2.500 | 23 | Pattern within height | |
| Landing | V-GRF | SOL vs SLJL | | High | 7–11% | | 0.00100 | 3.200 | 23 | Pattern within height | |
| Landing | V-GRF | SOL vs SLJL | | High | 20–33% | | <0.001 | 11.600 | 23 | Pattern within height | |
| Landing | V-GRF | SOL vs SLJL | | High | 34–45% | | <0.001 | 9.900 | 23 | Pattern within height | |
| Landing | V-GRF | High vs Low | | Within SLHL | 0–4% | | <0.001 | 3.200 | 23 | Height within pattern | |
| Landing | V-GRF | High vs Low | | Within SLHL | 6–13% | | <0.001 | 5.400 | 23 | Height within pattern | |
| Landing | V-GRF | High vs Low | | Within SLHL | 25–29% | | 0.00800 | 3.100 | 23 | Height within pattern | |
| Landing | V-GRF | High vs Low | | Within SLJL | 0–13% | | <0.001 | 12.400 | 23 | Height within pattern | |
| Landing | V-GRF | High vs Low | | Within SLJL | 23–87% | | <0.001 | 62.700 | 23 | Height within pattern | |
| Landing | V-GRF | High vs Low | | Within SOL | 0–6% | | <0.001 | 4.700 | 23 | Height within pattern | |
| Landing | V-GRF | High vs Low | | Within SOL | 7–17% | | <0.001 | 8.700 | 23 | Height within pattern | |
| Landing | V-GRF | High vs Low | | Within SOL | 23–71% | | <0.001 | 47.100 | 23 | Height within pattern | |
| Landing | AP-GRF | SLJL vs SLHL | | Low | 0–4% | | <0.001 | 3.700 | 23 | Pattern within height | |
| Landing | AP-GRF | SLJL vs SLHL | | Low | 6–8% | | <0.001 | 1.100 | 23 | Pattern within height | |
| Landing | AP-GRF | SLJL vs SLHL | | Low | 71–87% | | <0.001 | 14.400 | 23 | Pattern within height | |
| Landing | AP-GRF | SOL vs SLHL | | Low | 0–5% | | <0.001 | 3.800 | 23 | Pattern within height | |
| Landing | AP-GRF | SOL vs SLHL | | Low | 40–42% | | 0.00300 | 0.400 | 23 | Pattern within height | |
| Landing | AP-GRF | SOL vs SLJL | | Low | 1–5% | | <0.001 | 2.700 | 23 | Pattern within height | |
| Landing | AP-GRF | SOL vs SLHL | | High | 14–17% | | <0.001 | 2.200 | 23 | Pattern within height | |
| Landing | AP-GRF | SOL vs SLHL | | High | 32–52% | | <0.001 | 18.900 | 23 | Pattern within height | |
| Landing | AP-GRF | SOL vs SLHL | | High | 86–89% | | 0.00300 | 1.600 | 23 | Pattern within height | |
| Landing | AP-GRF | SOL vs SLJL | | High | 1–5% | | <0.001 | 3.400 | 23 | Pattern within height | |
| Landing | AP-GRF | SOL vs SLJL | | High | 41–46% | | 0.00100 | 4.300 | 23 | Pattern within height | |
| Landing | AP-GRF | High vs Low | | Within SLHL | 0–4% | | 0.00200 | 3.000 | 23 | Height within pattern | |
| Landing | AP-GRF | High vs Low | | Within SLHL | 13–22% | | <0.001 | 7.600 | 23 | Height within pattern | |
| Landing | AP-GRF | High vs Low | | Within SLJL | 0–5% | | <0.001 | 4.900 | 23 | Height within pattern | |
| Landing | AP-GRF | High vs Low | | Within SLJL | 10–12% | | 0.00400 | 0.600 | 23 | Height within pattern | |
| Landing | AP-GRF | High vs Low | | Within SLJL | 15–22% | | <0.001 | 6.300 | 23 | Height within pattern | |
| Landing | AP-GRF | High vs Low | | Within SOL | 0–6% | | <0.001 | 5.300 | 23 | Height within pattern | |
| Landing | AP-GRF | High vs Low | | Within SOL | 17–26% | | <0.001 | 8.000 | 23 | Height within pattern | |
| Landing | AP-GRF | High vs Low | | Within SOL | 79–96% | | <0.001 | 15.700 | 23 | Height within pattern | |
| Landing | AP-GRF | High vs Low | | Within SOL | 99–100% | | 0.00500 | 0.300 | 23 | Height within pattern | |
| Landing | ML-GRF | SOL vs SLHL | | Low | 31–35% | | 0.00100 | 2.900 | 23 | Pattern within height | |

Note: Cluster-level p values are supra-threshold cluster probabilities from paired SnPM comparisons. All comparisons used Npair = 23. Height codes are reported as High and Low, and movement initiation patterns are abbreviated as SOL (step-off landing), SLJL (single-leg jump landing), and SLHL (single-leg hop landing). Pattern-within-height comparisons form the primary post-hoc family and are displayed in Figures 2 and 3; height-within-pattern comparisons are reported here as a transparency check and are not plotted.

# Supplementary Results S1. Pattern Within Height SPM Cluster Summary

## Take-off Phase

Take-off phase SPM showed significant Pattern × Height interactions for V-GRF (60–89%), AP-GRF (0–2%, 3–6%, 7–15%, and 99–100%), and ML-GRF (27–32%). Within the primary pattern-within-height family, significant comparisons were present in five of six V-GRF contrasts, five of six AP-GRF contrasts, and one of six ML-GRF contrasts. The low-height SLJL–SLHL V-GRF difference at 78–92% was absent at the High height. Complete cluster locations and p values are reported in Supplementary Table S6.

## Landing Phase

Landing-phase SPM showed significant Pattern × Height interactions for V-GRF (8–10%, 12–18%, and 36–41%), AP-GRF (1–5% and 13–16%), and ML-GRF (14–16%). Within the primary pattern-within-height family, all six V-GRF contrasts, five of six AP-GRF contrasts, and one of six ML-GRF contrasts contained at least one significant cluster. Complete cluster locations and p values are reported in Supplementary Table S6.

# Supplementary Results S2. Height Within Pattern Secondary SPM Family

Height-within-pattern comparisons were treated as a secondary transparency family and were not plotted. Complete cluster-level results are reported in Supplementary Table S6.

Note: Intervals are percentages of the normalized take-off or landing phase. Height-within-pattern comparisons used Bonferroni-corrected α = 0.0167.

## Supplementary Figure S1. Associations between peak vertical ground reaction force (peak V-GRF) and (A) DPSI and (B) V-TTS across all valid trials (N = 276 trials from 23 participants).

**Note:** Each point represents one trial. Solid lines and shaded areas represent the fixed-effect estimate and 95% confidence interval from unadjusted linear mixed-effects models with participant as a random effect. In separate linear mixed-effects models fitted for each outcome, peak V-GRF was associated with DPSI (β = 0.049 BW⁻¹, 95% CI [0.046, 0.052], p < 0.001; marginal R² = 0.76, conditional R² = 0.86) and with V-TTS (β = 0.019 s·BW⁻¹, 95% CI [0.006, 0.032], p = 0.004; marginal R² = 0.02, conditional R² = 0.63). R² values shown in the panels are model-specific marginal R² values and were not statistically compared across outcomes. After adjustment for movement initiation pattern, height, and their interaction, the DPSI association remained positive (β = 0.030 BW⁻¹; 95% CI, 0.025 to 0.034; p < 0.001), whereas the V-TTS association changed direction (β = −0.051 s·BW⁻¹; 95% CI, −0.073 to −0.028; p < 0.001). DPSI = dynamic postural stability index; V-TTS = vertical time to stabilization; BW = body weight.

## Supplementary Table S7. Candidate time-to-stabilization (TTS) algorithms, threshold definitions, and operational stability criteria.

| **Processed signal** | **Direction** | **Threshold** | **TTS definition** | **Reference** |
| --- | --- | --- | --- | --- |
| SA | V, AP, ML | Trial mean ± 0.25 SD (0–5 s after contact) | Sequential average stays within bounds | 1 |
| TOP1 | V, AP, ML | Minimum fluctuation (10–20-s intervals) | First threshold intersection | 3,4 |
| TOP2 | AP, ML | Baseline variation mean (10–20 s) + 3 SD | First threshold intersection | 3,4 |
| TOP3 | AP, ML | Baseline variation mean (8–10 s) + 3 SD | First threshold intersection | 3,4 |
| RMS | AP, ML | Minimum fluctuation (10–20-s intervals) | Signal stays within bounds ≥0.5 s | 2 |
| WAV | V, AP, ML | Terminal 5-s wavelet energy ± 7 SD | Energy stays within bounds ≥0.5 s | 5 |

Note: SA = sequential averaging; TOP = unbounded third-order polynomial; RMS = root mean square; WAV = wavelet-based method; V = vertical; AP = anteroposterior; ML = mediolateral. Threshold definitions were algorithm- and direction-specific. For TOP2 and TOP3, mean refers to the mean baseline variation calculated within the specified time window. Reference numbers correspond to the Supplementary References. For SA, the sequential average remained within the bounds for the remainder of the 5-s post-contact window.

## Supplementary Table S8. Descriptive statistics and within-session between-trial repeatability of candidate TTS algorithms and stability-index outcomes across movement initiation patterns and drop height conditions.

| **Parameter** | **Height** | **SLHL** | | **SLJL** | | **SOL** | | **Pooled ICC[2,1] [95% CI]** |
| --- | --- | --- | --- | --- | --- | --- | --- | --- |
|  |  | **Mean ± SD** | **SEM** | **Mean ± SD** | **SEM** | **Mean ± SD** | **SEM** |  |
| APSI (Index) | Low | 0.068 ± 0.009 | 0.005 | 0.068 ± 0.008 | 0.005 | 0.071 ± 0.009 | 0.005 | 0.71 [0.59, 0.79] |
| APSI (Index) | High | 0.077 ± 0.008 | 0.005 | 0.078 ± 0.010 | 0.006 | 0.080 ± 0.009 | 0.005 | 0.71 [0.59, 0.79] |
| DPSI (Index) | Low | 0.315 ± 0.045 | 0.013 | 0.295 ± 0.047 | 0.014 | 0.236 ± 0.039 | 0.011 | 0.92 [0.87, 0.95] |
| DPSI (Index) | High | 0.391 ± 0.051 | 0.015 | 0.378 ± 0.059 | 0.017 | 0.337 ± 0.051 | 0.015 | 0.92 [0.87, 0.95] |
| MLSI (Index) | Low | 0.026 ± 0.006 | 0.004 | 0.031 ± 0.006 | 0.004 | 0.030 ± 0.006 | 0.004 | 0.62 [0.42, 0.73] |
| MLSI (Index) | High | 0.030 ± 0.007 | 0.005 | 0.033 ± 0.005 | 0.004 | 0.034 ± 0.008 | 0.006 | 0.62 [0.42, 0.73] |
| RMS AP (s) | Low | 0.77 ± 0.36 | 0.40 | 0.71 ± 0.45 | 0.39 | 0.68 ± 0.25 | 0.25 | 0.36 [0.18, 0.50] |
| RMS AP (s) | High | 0.94 ± 0.50 | 0.47 | 0.96 ± 0.48 | 0.48 | 0.76 ± 0.34 | 0.35 | 0.36 [0.18, 0.50] |
| RMS ML (s) | Low | 0.82 ± 0.28 | 0.30 | 0.81 ± 0.32 | 0.36 | 0.85 ± 0.17 | 0.22 | 0.22 [0.05, 0.42] |
| RMS ML (s) | High | 0.97 ± 0.30 | 0.42 | 0.87 ± 0.33 | 0.34 | 0.97 ± 0.52 | 0.58 | 0.22 [0.05, 0.42] |
| SA AP (s) | Low | 1.64 ± 0.38 | 0.25 | 2.25 ± 0.38 | 0.22 | 2.37 ± 0.27 | 0.17 | 0.71 [0.64, 0.76] |
| SA AP (s) | High | 1.57 ± 0.36 | 0.22 | 2.13 ± 0.29 | 0.18 | 2.26 ± 0.25 | 0.16 | 0.71 [0.64, 0.76] |
| SA ML (s) | Low | 2.32 ± 0.14 | 0.09 | 2.41 ± 0.16 | 0.11 | 2.63 ± 0.10 | 0.06 | 0.72 [0.56, 0.83] |
| SA ML (s) | High | 2.11 ± 0.17 | 0.10 | 2.20 ± 0.15 | 0.09 | 2.41 ± 0.15 | 0.09 | 0.72 [0.56, 0.83] |
| SA V (s) | Low | 1.98 ± 0.17 | 0.09 | 1.90 ± 0.17 | 0.08 | 1.82 ± 0.19 | 0.10 | 0.77 [0.64, 0.85] |
| SA V (s) | High | 1.96 ± 0.13 | 0.07 | 1.97 ± 0.13 | 0.07 | 1.90 ± 0.18 | 0.10 | 0.77 [0.64, 0.85] |
| TOP1 AP (s) | Low | 0.85 ± 0.76 | 0.65 | 1.09 ± 0.82 | 0.65 | 0.99 ± 0.72 | 0.59 | 0.49 [0.25, 0.67] |
| TOP1 AP (s) | High | 1.25 ± 0.88 | 0.75 | 1.30 ± 0.88 | 0.69 | 1.13 ± 0.82 | 0.70 | 0.49 [0.25, 0.67] |
| TOP1 ML (s) | Low | 2.35 ± 0.67 | 0.62 | 2.34 ± 0.63 | 0.56 | 2.61 ± 0.49 | 0.47 | 0.39 [0.22, 0.53] |
| TOP1 ML (s) | High | 2.65 ± 0.64 | 0.62 | 2.45 ± 0.59 | 0.54 | 2.65 ± 0.50 | 0.46 | 0.39 [0.22, 0.53] |
| TOP1 V (s) | Low | 3.23 ± 0.44 | 0.32 | 3.06 ± 0.54 | 0.39 | 2.73 ± 0.67 | 0.45 | 0.61 [0.42, 0.74] |
| TOP1 V (s) | High | 3.52 ± 0.38 | 0.30 | 3.39 ± 0.47 | 0.33 | 3.23 ± 0.58 | 0.40 | 0.61 [0.42, 0.74] |
| TOP2 AP (s) | Low | 0.72 ± 0.89 | 0.41 | 1.09 ± 1.03 | 0.45 | 1.07 ± 1.05 | 0.46 | 0.81 [0.71, 0.88] |
| TOP2 AP (s) | High | 1.10 ± 0.94 | 0.44 | 1.13 ± 0.92 | 0.42 | 1.20 ± 1.04 | 0.46 | 0.81 [0.71, 0.88] |
| TOP2 ML (s) | Low | 2.65 ± 0.69 | 0.30 | 2.51 ± 0.61 | 0.25 | 2.77 ± 0.60 | 0.25 | 0.83 [0.72, 0.89] |
| TOP2 ML (s) | High | 2.87 ± 0.71 | 0.31 | 2.66 ± 0.73 | 0.32 | 2.66 ± 0.74 | 0.31 | 0.83 [0.72, 0.89] |
| TOP3 AP (s) | Low | 0.89 ± 0.70 | 0.41 | 1.09 ± 0.52 | 0.27 | 1.01 ± 0.66 | 0.33 | 0.75 [0.58, 0.86] |
| TOP3 AP (s) | High | 1.26 ± 0.81 | 0.41 | 1.01 ± 0.65 | 0.34 | 1.27 ± 0.68 | 0.36 | 0.75 [0.58, 0.86] |
| TOP3 ML (s) | Low | 1.81 ± 0.30 | 0.24 | 1.72 ± 0.23 | 0.20 | 1.87 ± 0.26 | 0.20 | 0.45 [0.24, 0.75] |
| TOP3 ML (s) | High | 1.75 ± 0.40 | 0.33 | 1.88 ± 0.42 | 0.41 | 1.87 ± 0.33 | 0.28 | 0.45 [0.24, 0.75] |
| VSI (Index) | Low | 0.306 ± 0.045 | 0.013 | 0.285 ± 0.047 | 0.014 | 0.223 ± 0.039 | 0.011 | 0.92 [0.86, 0.95] |
| VSI (Index) | High | 0.382 ± 0.051 | 0.016 | 0.368 ± 0.060 | 0.017 | 0.325 ± 0.053 | 0.016 | 0.92 [0.86, 0.95] |
| WAV AP (s) | Low | 1.97 ± 0.71 | 0.94 | 2.04 ± 0.72 | 0.87 | 1.76 ± 0.61 | 0.81 | 0.08 [−0.04, 0.20] |
| WAV AP (s) | High | 2.03 ± 0.68 | 0.78 | 2.06 ± 0.75 | 0.96 | 2.31 ± 0.88 | 1.14 | 0.08 [−0.04, 0.20] |
| WAV ML (s) | Low | 1.27 ± 0.23 | 0.28 | 1.38 ± 0.21 | 0.29 | 1.44 ± 0.24 | 0.27 | 0.17 [0.01, 0.34] |
| WAV ML (s) | High | 1.17 ± 0.21 | 0.22 | 1.24 ± 0.18 | 0.21 | 1.36 ± 0.23 | 0.30 | 0.17 [0.01, 0.34] |
| WAV V (s) | Low | 1.44 ± 0.22 | 0.22 | 1.41 ± 0.20 | 0.20 | 1.42 ± 0.24 | 0.20 | 0.38 [0.13, 0.59] |
| WAV V (s) | High | 1.31 ± 0.22 | 0.21 | 1.31 ± 0.26 | 0.23 | 1.32 ± 0.26 | 0.26 | 0.38 [0.13, 0.59] |

Note: Data are presented as mean ± SD. ICC = intraclass correlation coefficient; CI = confidence interval; SEM = standard error of measurement; TTS = time to stabilization; SI = stability index; DPSI = dynamic postural stability index; APSI = anteroposterior stability index; MLSI = mediolateral stability index; VSI = vertical stability index; V = vertical; AP = anteroposterior; ML = mediolateral; SA = sequential averaging; TOP = unbounded third-order polynomial; RMS = root mean square; WAV = wavelet-based method; SOL = step-off landing; SLJL = single-leg jump landing; SLHL = single-leg hop landing. Low and High indicate the two individualized drop height conditions. Mean ± SD values are based on each participant’s two-trial condition mean. SEM was calculated for each condition as the condition-specific trial-level standard deviation multiplied by √(1 − pooled ICC[2,1]). The pooled ICC[2,1] for each outcome was estimated across all participant × pattern × height targets and is therefore identical across conditions within an outcome. ICC 95% CIs were obtained from 5,000 participant-level bootstrap samples, resampling participants with replacement and retaining all available complete targets and both trials from each sampled participant. The pooled ICC was used as a common reliability coefficient for the condition-specific SEM calculations; therefore, the resulting SEM values are approximate and should not be interpreted as separate condition-level reliability estimates. Between-condition differences may contribute to the pooled variance.

# Supplementary References

1. Colby SM, Hintermeister RA, Torry MR, Steadman JR. Lower limb stability with ACL impairment. J Orthop Sports Phys Ther. 1999;29:444-451.

2. Tulloch E, Phillips C, Sole G, Carman A, Abbott JH. DMA clinical Pilates directional-bias assessment: reliability and predictive validity. J Orthop Sports Phys Ther. 2012;42:676-687.

3. DiStefano LJ, Padua DA, Blackburn JT, Garrett WE, Guskiewicz KM, Marshall SW. Integrated injury prevention program improves balance and vertical jump height in children. J Strength Cond Res. 2010;24:332-342.

4. Kaminski TW, Ross SE, Guskiewicz KM. Time to stabilization: a method for analyzing dynamic postural stability. Athl Ther Today. 2003;8:37-39.

5. García-Massó X, Skypala J, Jandacka D, Estevan I. Reliability of a new analysis to compute time to stabilization following a single leg drop jump landing in children. PLoS One. 2019;14:e0212124.

6. Byrne A, Lodge C, Wallace J. Test-retest reliability of single-leg time to stabilization following a drop-landing task in healthy individuals. J Sport Rehabil. 2021;30:1242-1245.

7. Fransz DP, Huurnink A, de Boode VA, Kingma I, van Dieën JH. Time to stabilization in single leg drop jump landings: an examination of calculation methods and assessment of differences in sample rate, filter settings and trial length on outcome values. Gait Posture. 2015;41:63-69.

8. Fransz DP, Huurnink A, de Boode VA, Kingma I, van Dieën JH. The effect of the stability threshold on time to stabilization and its reliability following a single leg drop jump landing. J Biomech. 2016;49:496-501.

9. Wikstrom EA, Tillman MD, Smith AN, Borsa PA. A new force-plate technology measure of dynamic postural stability: the Dynamic Postural Stability Index. J Athl Train. 2005;40:305-309.
